# “Friends” that come with man’s best friend: *Capnocytophaga canimorsus* sepsis and meningitis in an immunocompromised patient

**DOI:** 10.1128/asmcr.00085-24

**Published:** 2025-02-14

**Authors:** Lili Tao, Jeffrey A. Freiberg, Romney Humphries

**Affiliations:** 1 Division of Laboratory Medicine, Department of Pathology, Microbiology and Immunology, Vanderbilt University Medical Center, Nashville, Tennessee, USA; 2 Division of Infectious Diseases, Department of Medicine, Vanderbilt University Medical Center, Nashville, Tennessee, USA; Pattern Bioscience, Austin, Texas, USA

**Keywords:** *Captocytophaga canimorsus*, sepsis, meningitis, molecular methods

## Abstract

**Background:**

*Capnocytophaga canimorsus* is a commensal bacterium found in the normal oral microbiota of dogs and cats. It can cause severe systemic infections, such as bacteremia and meningitis, in both immunocompromised and immunocompetent humans.

**Case Summary:**

We present a case of *C. canimorsus* bacteremia and meningitis with fever, headache, and altered mental status, which posed diagnostic challenges due to the administration of antimicrobials prior to specimen collection.

**Conclusion:**

This case highlights the value of molecular methods in diagnosing severe infections caused by fastidious bacteria and in cases of antibiotic use before culture.

## INTRODUCTION

*Capnocytophaga canimorsus* is a capnophilic, small Gram-negative rod that is a commensal resident of the oral microbiota of dogs, cats, and a small proportion of humans. *C. canimorsus* has been reported to cause life-threatening infections in humans, including bacteremia, meningitis, infectious aneurysm, and endocarditis (1, 2). Isolation and identification of this bacterium is technically challenging due to its fastidious nature, which may result in substantial delay in diagnosis. Here, we present a case of severe sepsis due to bacteremia and meningitis caused by *C. canimorus*, highlighting the value of molecular testing in such infections.

## CASE PRESENTATION

A 76-year-old man with a past medical history notable for hypertension, chronic artery disease, type 2 diabetes mellitus, and diffuse large B-cell lymphoma in remission was initially brought to a local emergency department (ED) by his family for evaluation and treatment of altered mental status and hypoxia after he was found unresponsive at home. The patient’s family noted that the patient had a fever and headache for 2 days prior to presentation. In the local ED, a blood culture was collected, and the patient received intravenous (IV) vancomycin and piperacillin/tazobactam prior to transfer to our institution. Upon arrival to the ED of our institution (day 0), two new sets of blood cultures (each set of blood cultures consists of one BACTEC Aerobic PLUS bottle and one BACTEC Lytic/Anaerobic bottle, BD, Sparks MD) were collected and he was empirically started on vancomycin 1,250 mg daily IV, ceftriaxone 2 g every 12 hours IV, ampicillin 2 g every 4 hours IV, acyclovir 10 mg/kg every 8 hours IV, and doxycycline 100 mg every 12 hours IV.

**Fig 1:**
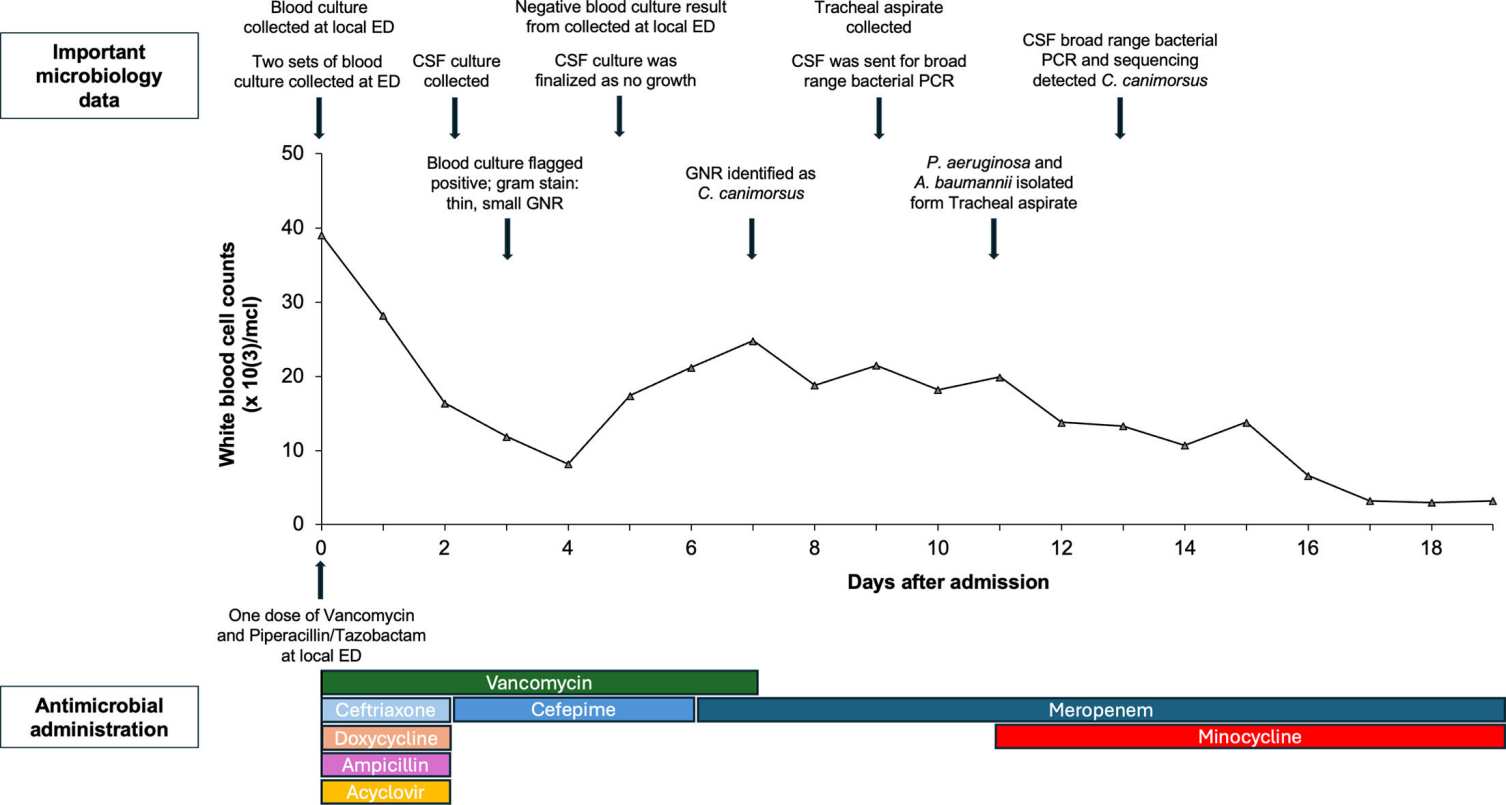
Dynamic of white blood cell counts (middle), important microbiology specimen collection and results (top), and antimicrobial administration (bottom) of the patient. ED, emergency department; CSF, cerebrospinal fluid; GNR, Gram-negative rod.

**Fig 2:**
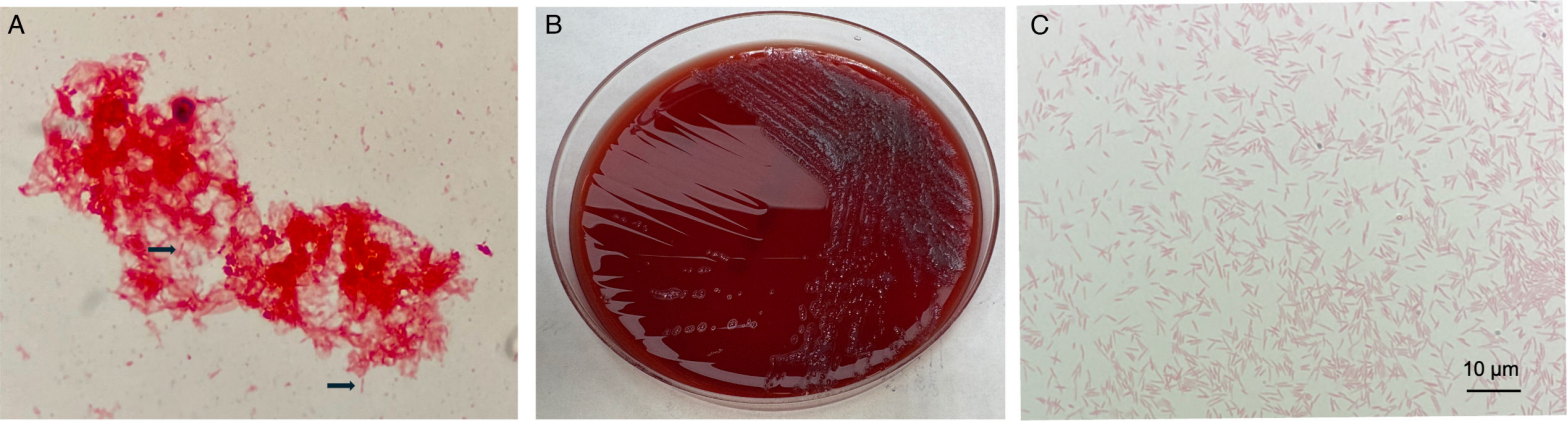
Morphology of *Capnocytophaga canimorsus*. (**A**) Gram stain of blood culture showed thin, elongated Gram-negative rods (black arrow). (**B**) Culture of *Capnocytophaga canimorsus* on blood agar showed small flat colonies with spreading edges. (**C**) Gram stain of *Capnocytophaga canimorsus* from colonies.

Initial complete cell count (CBC) demonstrated significant leukocytosis at 39.1 × 10^3^ cells/mcl (Fig. 1), with neutrophils as the dominant cell type (absolute neutrophil count: 36.1 × 10^3^ cells/mcl). On day 3, one aerobic blood culture bottle (BD BACTEC Plus, Sparks MD) from two sets of blood cultures (four bottles) collected at our ED flagged positive on a BD BACTEC FX instrument. Gram stain demonstrated thin, small Gram-negative rods (Fig. 2) and molecular testing using Verigene Gram-Negative Blood Culture test (Luminex, TX, USA) was unable to identify the organism. The positive blood culture was subcultured to Sheep’s blood agar (SBA), chocolate agar (CHOC), and MacConkey and incubated aerobically in 5% CO_2_ at 35°C. The media were selected based on the laboratory’s standard operating procedure to enable the growth of most Gram-negative rods, including fastidious isolates that may not grow on SBA or MacConkey. The laboratory routinely incubates subcultures from blood specimens in 5% CO_2_ to facilitate the recovery of capnophilic bacteria, which require CO_2_ to support their growth. On day 7, a thin film of bacterial growth was visible on SBA and CHOC, with no growth on the MacConkey agar. The identification of *C. carnimorsus* was ultimately obtained 7 days after admission by matrix-assisted laser desorption ionization-time of flight mass spectrometry (MALDI-TOF MS, Microflex LRF, Bruker Daltonics, Billerica, Massachusetts) with a score of 2.39 from the Biotyper research use only library. The other three blood culture bottles collected at our ED were finalized as having no growth after 5 days of incubation. The blood culture collected at the local ED was also reported as having no growth after 5 days of incubation.

Although the patient presented with altered mental status, a lumbar puncture was delayed due to the patient’s use of dual antiplatelet therapy (aspirin and clopidogrel). Brain magnetic resonance imaging revealed extensive meningitis and ventriculitis, without evidence of acute infarction or lesion. A lumbar puncture on hospital day 2 revealed an opening pressure of 34 cm H_2_O and cloudy cerebrospinal fluid (CSF). CSF analysis showed 2,374 cells/µL nucleated cells with 94% neutrophils. CSF glucose was elevated (91 mg/dL, reference range: 45–71 mg/dL) as was protein (223 mg/dL, reference range: 15–40 mg/dL). Gram stain of CSF concentrated by cytospin revealed a significant number of nucleated cells, with no bacteria observed. Molecular testing of the CSF using the FilmArray meningitis/encephalitis panel (BioFire Diagnostics, Utah, USA) was negative. Bacterial cultures performed on the CSF were also negative. CSF collected on day 2 was sent to the University of Washington for broad-range bacterial PCR followed by sequencing on day 9 of admission, which detected *C. canimorsus* DNA on day 13 of admission.

### Antimicrobial treatment

Acyclovir and doxycycline were discontinued on day 2 of admission, following the results of the CSF analysis and molecular testing. However, the patient was escalated to vancomycin 1,250 mg daily IV and meropenem 2 g every 8 hours on day 6 after admission due to continued fevers and relapsed leukocytosis (Fig. 1). On hospital day 7, antimicrobials were narrowed to meropenem (2 g every 8 hours) monotherapy after the identification of *C. carnimorsus* in the blood cultures. The patient’s clinical course was further complicated by a ventilator-associated respiratory infection. A tracheal aspirate culture collected on day 9 of admission grew a pan-susceptible *Pseudomonas aeruginosa* and a multidrug-resistant *Acinetobacter baumannii*. These were treated with minocycline 100 mg twice daily through a feeding tube, in addition to the meropenem used to treat the *C. carnimorsus* infection. The patient’s fevers, encephalopathy, and respiratory status ultimately improved with antimicrobial treatment, and he was extubated on day 14 of admission. The patient continued on meropenem for a total of 3 weeks and completed a 1 week course of minocycline. He was discharged from the hospital after 4 weeks with significant diffuse weakness and oropharyngeal dysphagia due to polyneuropathy from critical illness. The patient agreed to share information for the case report, and informed consent has been obtained.

## DISCUSSION

*C. canimorsus* causes zoonotic infections associated with dog bites, and less frequently, cat bites (3–5). It is now recognized that a bite is not essential for infection as many cases of sepsis and meningitis caused by this organism were not associated with a dog bite, but close contact with dogs has been frequently reported (1, 3). In our case, the patient denied any history of a dog bite and no evidence of a bite wound was identified. Upon identification of the blood isolate, however, it was determined he owned a 6-month-old Australian Shepherd. Interestingly, recent reports suggest transmission of *C. canimorsus* from other animals, including diverse sources such as a lion bite and the large pine weevil, *Hylobius abietis* (6, 7).

*C. canimorsus* meningitis has classically been characterized as a disease of immunocompromised patients, particularly asplenic patients. However, this risk factor accounts for a small proportion of cases (3). Other risk factors for developing invasive *C. canimorsus* infections include alcohol use, liver cirrhosis, smoking, and malignancy. It is worth noting that severe infections of *C. canimorsus* in otherwise immunocompetent patients have also been reported (8–11). Our patient’s medical history was notable for large B-cell lymphoma, which was in remission, and several chronic medical conditions including type 2 diabetes mellitus, which may have heightened his risk for this infection. Data documenting the routine antimicrobial susceptibility patterns of *C. canimorsus* are sparse*,* although in general these organisms display reduced susceptibility (i.e., elevated MICs) to aminoglycosides, trimethoprim, and metronidazole and lower MICs to beta-lactams, clindamycin, and the fluoroquinolones (3, 12). Beta-lactams are typically the antimicrobials of choice, although an increasing incidence of extended-spectrum beta-lactamase-producing isolates has been reported in *Capnocytophaga* spp. other than *C. canimorsus* (13, 14).

The diagnosis of *C. canimorsus* infection is challenging due to the organisms’ fastidious and slowly growing nature. In our case, it took 3 days to grow in the blood culture bottle, and an additional 4 days to grow on subculture to SBA. The antimicrobials administered to the patient prior to the collection of blood cultures and lumber puncture are likely contributing factors to its slow growth in the blood culture and lack of growth in CSF. However, the morphology of the bacteria from a Gram stain and a negative Verigene Gram-Negative Blood Culture test (including *Escherichia coli*, *Klebsiella oxytoca*, *Klebsiella pneumoniae*, *Pseudomonas aeruginosa*, *Acinetobacter* spp., *Citrobacter* spp., *Enterobacter* spp., and *Proteus* spp. as bacterial targets) indicated possible *Capnocytophaga* spp., *Fusobacterium nucleatum,* and *Leptotrichia* spp., although *Fusobacterium nucleatum* is an anaerobe that usually doesn’t grow in aerobic bottle. Biochemical tests that might distinguish zoonotic *C. canimorsus* and *C. cynodegmi* from the other *Capnocytophaga* species include a positive catalase test and a positive oxidase test (all other *Capnocytophaga* species are catalase-negative and oxidase negative). Notably, two sets of blood cultures collected at a local ED, prior to the administration of any antibiotics, were finalized as no growth after 5 days. This may have been due to insufficient volume collected for blood culture at the local ED, or intermittent bacteremia, although no further details regarding that blood collection could be obtained. Identification of *Capnocytophaga* species using MALDI-TOF MS has been challenging due to the limited reference library. *C. canimorsus* was not considered a clinically validated species in the MALDI Biotyper CA library in the United States until 2021, while it is still not included in the VITEK MS database (V3.3, bioMérieux, Marcy-l'Étoile, France). However, the identification of *Capnocytophaga* species using MALDI-TOF MS is reliable if a species-level score is achieved (15, 16).

Case reports of invasive *C. canimorsus* infections have increased significantly in the past 2–3 years, partly due to the use of molecular detection methods, such as polymerase chain reaction (PCR) and next-generation sequencing of cell-free DNA from blood (3, 17, 18). Molecular methods are extremely helpful in diagnosing cases of *C. canimorsus* endocarditis which are otherwise negative with tissue culture (19–22). However, these tests are not available in most clinical microbiology laboratories due to technical challenges and the lack of commercial assays. As a result, these assays are typically offered as second-tier tests when conventional cultures are negative. Additionally, there may be further delays in obtaining results due to the need for specimen transportation from clinical centers to reference laboratories. In our case, although we successfully isolated *C. canimorsus* from blood culture, it took almost a week to achieve the final identification of the organism. In addition, the CSF culture of our patient was negative, and the final diagnosis of *C. canimorsus* meningitis was made based on 16S rRNA gene PCR and subsequent sequencing. Two recent case reports and literature reviews summarized the advantage of PCR and next-generation sequencing for the diagnosis of *C. canimorsus* meningitis, including many cases diagnosed only through molecular tests (3, 17). It therefore emphasizes the importance of using molecular methods for the diagnosis of infectious diseases caused by fastidious bacteria and in cases of antibiotic use before specimen collection.

In conclusion, we report a case of *C. canimorsus* sepsis and meningitis in an immunocompromised patient with close contact with an Australian Shepherd puppy. The diagnosis of the pathogen from blood culture was delayed due to its fastidious and slow-growing nature, and the identification of the pathogen from CSF was achieved only with the aid of 16S rRNA PCR and subsequent sequencing. Molecular methods including 16S rRNA PCR or metagenomic sequencing are useful for the accurate diagnosis of serious infections, especially when associated with fastidious bacterial infections and in cases of antibiotic use before culture.
